# Ohio's Dual Eligible Population: Effects of Program Design and Implementation on Care Management

**DOI:** 10.1093/geroni/igab046.1749

**Published:** 2021-12-17

**Authors:** Jennifer Heston-Mullins, Katherine Abbott, Athena Koumoutzis

**Affiliations:** Miami University, Oxford, Ohio, United States

## Abstract

MyCare Ohio is a prospective blended managed care payment model program tasked to provide comprehensive and coordinated care to Ohio residents who are dully eligible for Medicare and Medicaid. To understand the administration and day-to-day implementation of care management within MyCare Ohio, n=75 interviews with a total of n=331 personnel from Area Agencies on Aging, Managed Care Plans, and service providers were conducted. Interviews were audio recorded, transcribed, and checked for accuracy. Data were analyzed by iterative reviews and deductive coding in Dedoose. Respondents provided insights on how care management activities are affected by program design features (e.g., ability to opt-out of the Medicare component), transitions between acute and long-term care settings, documentation systems and data-sharing, and high numbers of beneficiaries with behavioral health diagnoses. Implications for practice and policy will be discussed.

